# Dengue prediction by the web: Tweets are a useful tool for estimating and forecasting Dengue at country and city level

**DOI:** 10.1371/journal.pntd.0005729

**Published:** 2017-07-18

**Authors:** Cecilia de Almeida Marques-Toledo, Carolin Marlen Degener, Livia Vinhal, Giovanini Coelho, Wagner Meira, Claudia Torres Codeço, Mauro Martins Teixeira

**Affiliations:** 1 Departamento de Bioquimica e Imunologia do Instituto de Ciencias Biologicas, Universidade Federal de Minas Gerais, Belo Horizonte, Minas Gerais, Brazil; 2 Consultoria Tecnica, Ecovec LTDA, Belo Horizonte, Minas Gerais, Brazil; 3 Programa de Computacao Cientifica, Fundacao Oswaldo Cruz, Rio de Janeiro, Rio de Janeiro, Brazil; 4 Secretaria de Vigilancia em Saude, Ministerio da Saude, Brasilia, Brazil; 5 Departamento de Ciencia da Computacao do Instituto de Ciencias Exatas, Universidade Federal de Minas Gerais, Belo Horizonte, Minas Gerais, Brazil; Institute for Disease Modeling, UNITED STATES

## Abstract

**Background:**

Infectious diseases are a leading threat to public health. Accurate and timely monitoring of disease risk and progress can reduce their impact. Mentioning a disease in social networks is correlated with physician visits by patients, and can be used to estimate disease activity. Dengue is the fastest growing mosquito-borne viral disease, with an estimated annual incidence of 390 million infections, of which 96 million manifest clinically. Dengue burden is likely to increase in the future owing to trends toward increased urbanization, scarce water supplies and, possibly, environmental change. The epidemiological dynamic of Dengue is complex and difficult to predict, partly due to costly and slow surveillance systems.

**Methodology / Principal findings:**

In this study, we aimed to quantitatively assess the usefulness of data acquired by Twitter for the early detection and monitoring of Dengue epidemics, both at country and city level at a weekly basis. Here, we evaluated and demonstrated the potential of tweets modeling for Dengue estimation and forecast, in comparison with other available web-based data, Google Trends and Wikipedia access logs. Also, we studied the factors that might influence the goodness-of-fit of the model. We built a simple model based on tweets that was able to ‘nowcast’, i.e. estimate disease numbers in the same week, but also ‘forecast’ disease in future weeks. At the country level, tweets are strongly associated with Dengue cases, and can estimate present and future Dengue cases until 8 weeks in advance. At city level, tweets are also useful for estimating Dengue activity. Our model can be applied successfully to small and less developed cities, suggesting a robust construction, even though it may be influenced by the incidence of the disease, the activity of Twitter locally, and social factors, including human development index and internet access.

**Conclusions:**

Tweets association with Dengue cases is valuable to assist traditional Dengue surveillance at real-time and low-cost. Tweets are able to successfully *nowcast*, i.e. estimate Dengue in the present week, but also *forecast*, i.e. predict Dengue at until 8 weeks in the future, both at country and city level with high estimation capacity.

## Author summary

Dengue is a fast-growing mosquito-borne viral disease, with an estimated annual incidence of 390 million infections, of which 96 million manifest clinically. Dengue burden is likely to increase in the future. Mentioning a disease in social networks is correlated with physician visits by patients, and can be used to estimate disease activity. Traditional, biologically-focused monitoring techniques, based on laboratory diagnostics, are accurate but costly and slow. Alternative approaches for surveillance aim to capture health-seeking behavior at earlier stages of disease progression, specially capturing the asymptomatic and mild clinic manifestation population who do not seek medical care formally. Twitter data have potential application for Dengue surveillance, improving the estimation and prediction of the disease, in space and time, being a valuable and low-cost addition to assist traditional surveillance. We show that tweets are strongly associated with Dengue cases. Tweets are a useful tool for estimating and forecasting Dengue cases until 8 weeks in the future, both at country and city level, even in less developed areas.

## Introduction

Infectious diseases are a leading threat to public health, economic stability, and other key social structures [1]. Efforts to mitigate these impacts depend on accurate and timely monitoring to measure the risk and incidence of the disease [2,3,4]. Early detection of disease activity and rapid responses can reduce the impact of diseases [5]. The interdisciplinary field of computational social science aims to quantify real-world social phenomena using large datasets known as ‘big data’, based on data from social networks, such as Twitter and Facebook, to describe behavioral patterns in novel contexts [6,7]. The relative frequency of mentioning a disease in certain social networks, as in Twitter and others, is highly correlated with patients visits to doctors, making it possible to accurately estimate disease activity in each region of a country, with a small reporting lag [3,5]. Also, web search query data from Google and Wikipedia are capable of tracking disease activity and are available in near real-time [2,8].

Twitter is a unique social media channel, since users inform and discuss, through their short 140-character messages or ‘tweets’, about the most diverse topics, including health conditions [3,9,10]. This free social networking service has more than 190 million users registered worldwide and processes about 55 million tweets per day, with the possibility of sentiment analysis and location selection [3,9,10,11]. According to the US Bureau (2014), 45% of the Brazilian households have access to internet and 48% of the population are users of social media products, with an average of 3 hours per day spent in this activity. In Brazil, the main social media of preference is Facebook (94%), followed by Google Plus (75%), Twitter (56%) and LinkedIn (54%) [12]. Important to notice that Twitter usage (86%), as of other social media products, is mostly via mobile phone, which in Brazil has 134% of coverage, meaning an average of 1.34 cell phone subscriptions per person [12].

Dengue is an important public health burden, likely to increase in the future due to increased urbanization, scarce water supplies and environmental change [13]. Dengue is ubiquitous throughout the tropics and a fast spreading viral mosquito-borne disease [14], with an incidence increase of 30 times during the last 50 years [15]. The World Health Organization (WHO) estimates 50 to 100 million new infections per year in 100 different countries, with half the world’s population, or 3.5 billion people at risk [15], but more recent studies estimate the total incidence to be 390 million Dengue infections per year, of which 96 million manifest clinically [14]. Currently, there is no specific antiviral treatment to reduce severe illness or an effective vaccine to induce strong protection from infection [16]. The epidemiology of Dengue in Brazil is characterized by increasing geographical spread, as well as the total incidence of reported cases [17]. The epidemiological dynamics of Dengue disease is complex, and difficult to predict, partly due to the weaknesses of passive surveillance systems [13,17]. The majority of infections are clinically non-specific, consequently, Dengue disease is often underdiagnosed [13], but these patients are also infectious to mosquitoes and contribute for the transmission of the disease [18].

Bureaucracy and lack of resources have interfered with timely detection and reporting of Dengue cases in many endemic countries, including Brazil, where reporting delay is estimated to be of 3 to 4 weeks [3, 19]. Traditional, laboratory and clinically based diagnostic techniques are accurate but costly and slow. Alternative approaches to surveillance aim to capture health-seeking behavior at earlier stages of disease progression, specially capturing those with mild clinic manifestation population who do not seek medical care formally [2,6]. Some studies indicate that digital media reports reflect national epidemiological trends, acting as proxy for surveillance to provide early warning and situation awareness of emerging infectious diseases and Dengue [4,20]. While traditional Dengue surveillance data suffer from substantial delay, web-based data can fill in the gap providing a near real-time source of information [8,21,22]. Previous studies have shown that Twitter is a real-time source of information on Dengue symptoms activity in a population, and shows strong correlation with the number of notified cases [3, 23]. Some advocate that Twitter-based surveillance efforts may provide an important and cost-effective supplement to traditional disease-surveillance systems [10,11]. Besides Twitter, web search query data, such as Google Trends, were also found to be capable of tracking Dengue activity. Proper combination of these two sources of information may provide timely information to public health officials and contribute to real-time predictive models [8, 23]

In this study, we aimed at investigating if tweets with personal indication of Dengue content could be integrated with clinical Dengue data to produce an accurate model for the early detection and monitoring of Dengue epidemics at country and city level. For comparison, we also report other available web-based data, Google Trends and Wikipedia. Also, we studied the factors that might influence the goodness-of-fit of the proposed model. We concluded that a simple model using tweets is able to successfully nowcast, i.e. estimate Dengue in the present week, and forecast, i.e. predict Dengue until 8 weeks in the future, both at country and city level with good estimation capacity. Our model can be applied successfully to smaller and less developed cities, even though it may be influenced by the incidence of the disease, the activity of Twitter locally, and social factors, including human development index and internet access.

## Methods

### Data sources

#### Official Dengue case data

Reported Dengue cases were obtained from the Brazilian Ministry of Health, at the SINAN-net (Sistema de Informação de Agravos de Notificação), available from: http://portalweb04.saude.gov.br/sinan_net/default.asp. The majority of cases were ascertained using clinical-epidemiological criteria. We considered the date of symptoms onset as the date of the disease to construct the weekly time series of Dengue incidence, which was calculated counting suspected and confirmed cases, excluding discarded cases, aggregated by city and week, between September, 2012 and October, 2016. Data aggregation per week followed the epidemiological week definition, where week one of every year is the first week with 4 days or more. If the first week has 3 days or less it is accounted as the last week of the previous year. Each year has 52 or 53 weeks.The Dengue transmission season in Brazil occurs primarily between the months of November and April, hence to consider a full disease transmission cycle, we defined the epidemiological year as the period having November as month 1, December as month 2, and so on. We also transformed the standard weeks 1 to 52 (or 53) per year, in weeks per epidemiological year (*e*year), starting in the first week of November.

#### Tweets

The Observatorio da Dengue Lab (ODL, available at: www.observatorio.inweb.org.br/Dengue/) has a web-service that monitors the twitting activity associated with Dengue in Brazil. The method consists in collecting all tweets containing one of the key words: “Dengue”, “aedes” and “aegypti”, then, a machine learning algorithm was developed to identify and select only those suggestive of a personal experience with Dengue (i.e. being infected, knowing someone with Dengue, and others). This is an automatic classifier, trained based on previous classifications performed by human specialists, as described in [3,9]. Other categories, besides personal experience, are: parody, opinion, information and marketing campaigns. The dataset contains: the total number of tweets with Dengue content captured per unit of time and space; the geographic information associated with the Twitter user; the time when the tweet was posted; and the content, according to the aforementioned classification. Tweets are allocated to the city they were most likely posted from. This is preferentially done by using the location provided by the device’s gps (geographic position system) and stored by Twitter at the moment the message was posted. If the device’s gps was disabled, the most likely location is drawn from the address informed by the user during his registration at Twitter. The total number of messages and unique users since 2012, when the application was started, is listed in Table 1. The data refer to the total number of tweets with Dengue content before classification into personal experience. In Table 1, we can observe that 2014 has the smallest number of users and messages, probably associated with a reduced number of Dengue cases (59% less than 2013) [24]. In this study, we analyzed the Twitter activity of a set of 283 cities in Brazil, encompassing cities monitored by the *Observatorio*, with an average of at least 1 tweet posted (Dengue with all contents) per day during three following weeks.

**Table 1:** General description of Dengue activity for Twitter dataset in Brazil (Kind et al, 2016).

Data	2012	2013	2014	2015	2016	Total
Twitter messages with Dengue content (n)	303,102	285,823	177,093	417,882	475,461	1,659,361
Unique users posting Dengue tweets (n)	147,447	128,260	816,44	145,435	150,911	653,697
% of georefered messages	63	72	74	74	74	69
Cities	2,826	2,991	2,598	3,131	3,168	4,597

#### Google trends

In this study, the main source of media data is the Twitter, due to its greater expressiveness (that is, users are authors of their messages). Still, other sources of digital data have been advocated as sensitive indicators of Dengue activity, among them, Google Trends. Google search queries containing the word “Dengue” were obtained for Brazil from 2012 to 2016 and aggregated per week. Data are available at country and state level. Here we considered only country level data. At Google Trends, online anonymized logs submitted since 2004 is computed as a relative measure of the online search queries submitted in that country on that week. The measure represents the relative interest for this topic in relation to the highest peak of interest for this same topic over the period analyzed. Our data request was carried out in December, 18^th^, 2016, comprising the complete dataset for Brazil since January 2004. The data are freely available at the Google Trends website (https://www.google.com/trends/explore#q=dengue). No information about the identity of any user was retained, including IP address, once the country of origin was determined (http://www.google.com/privacy/privacy-policy.html).

#### Wikipedia article access logs

For comparison, we further considered the time series of digital activity related to Dengue provided by visits to Wikipedia. Access logs for all Wikipedia articles were obtained from the web interface http://stats.grok.se, where these logs are available with language and time period selection. Data is available per language and have no direct reference to country or region. These data encompass a variety of activities, including article views, article visits and page views. Our data request was performed with the single keyword “Dengue” at December, 18^th^, 2016, but data are available only until December, 2015. We computed the daily and weekly number of searches for the word “Dengue” with language selection as “Portuguese” or the web interface wikipedia.com/br. Data was then aggregated per week according to epidemiological week.

#### Sociodemographic data

In order to analyze factors that might influence the Dengue estimation capacity of the tweets models, we evaluated a variety of sociodemographic indices. From the 2010 census (Brazilian Institute for Geography and Statistics) [25], we obtained the following indices: population, per capita GDP (gross domestic product), mean human development index (IDHM), income human development index (IDHM-Income), education human development index (IDHM-Education), and longevity human development index (IDHM-Longevity). We also evaluated the percentage of houses with internet access, and with personal computer, as available elsewhere [26].

### Statistical analyses

#### Levels of analysis

We analyzed the data at city level (283 Brazilian cities with population over 40,000 inhabitants and Twitter activity) and at country level (by summing all Dengue cases and Dengue-related tweets for the country).

#### Correlation between Dengue and web-based data at country level

Evidence of association between Dengue cases and web-based data from Twitter, Google Trends, and Wikipedia access logs were investigated at national level by plotting their time series and assessing their linear regression.

#### Model fitting

Only Twitter data with Dengue content were selected for further exploratory analysis and model fitting at city and country-level, since Google Trends and Wikipedia are not available at city resolution [2,5,6,8,22]. For this purpose, tweets were considered the explanatory variable and Dengue cases the response variable. Preliminary analyses indicated a non-linear association between tweets and Dengue. To model the association between tweets and Dengue, we used a generalized additive model (GAM) of the form:

$$\begin{array}{c} Dengue_{t}∼\mathrm{Negative}\;\mathrm{Binomial}\;\left(\mu_{t},\;\kappa \right)\\ \log\left(\mu_{t}\right)={ƒ}_{1}\left(tweets_{t}\right)+{ƒ}_{2}\left(week_{t}\right)+eyear_{t}+\beta 0 \end{array}$$

where log(*μ*_t_) is the logarithm of the number of Dengue cases in week *t* (*t* = 1,…, 209), estimated based on the smooth spline of tweets, ƒ_1_(*tweets*_t_), which indicates the number of tweets with Dengue content in that same week *t*; ƒ_2_(*week*_t_) is the smooth spline of the epidemiological week (*week* = 1,…, 53); and e*year* is a factor for each epidemiological year (e*year* = 2012,…, 2015); *β*0 is the intercept, and *κ* is the dispersion parameter.

A negative binomial model was necessary to account for the over-dispersion of the response variable (Dengue case counts per week). This model was compared to simpler versions (without the nonlinear terms) and more complicated versions (i.e. models with interaction terms between week and year, and autocorrelation terms (AR-1)) (S1 Fig). Goodness-of-fit was evaluated using the Akaike Information Criterion (AIC) [27], as well as diagnostic residual plots and by plotting the observed versus predicted values. The same modeling procedure was repeated for each of the 283 cities included in the study, but the model could successfully fit an estimate in 199 of them. In most cases, the above-mentioned model was the most adequate.

To evaluate model estimation and goodness-of-fit, we considered their deviance (Explained deviance = 1 –(residual deviance/null deviance)), adjusted correlation index (r^2^), and also the mean percentage relative error, calculated as the mean relative difference between predicted Dengue cases and observed cases. All analyses were performed in R version 3.2.3 (2015/12/10, Vienna, Austria), using the packages: ggplot2 [28]; lattice [29]; scales [30]; mgcv [31]; and ggmap [32].

#### Out-of-sample validation analyses

For the model validation, the datasets were divided into training and validation sets. The training set, used for model fitting, included the first 170 weeks of the dataset, while the validation sets included the remaining 39 weeks’ time series.

#### Forecast analyses

In order to evaluate if tweets are useful for Dengue forecasting, we fitted the following model with a sequence of delays:

$$\begin{array}{c} Dengue\left(t\right)∼\mathrm{Negative}\;\mathrm{Binomial}\;\left(\mu_{t},\;\kappa \right)\\ \log\left(\mu_{t}\right)={ƒ}_{1}\left(tweets_{t\text{-}x}\right)+{ƒ}_{2}\left(week_{t}\right)+year_{t}+\beta 0 \end{array}$$

where the number of Dengue cases in week *t* are predicted based on the number of tweets from *x* weeks before Dengue cases occurrence. We tested this model with different time lags, with *x* varying from 1 to 8 weeks and report the deviance explained index, AIC value and the mean relative error for each time lag.

## Results

### Tweets and other web-based data are associated with Dengue cases at country level

**Fig 1:**
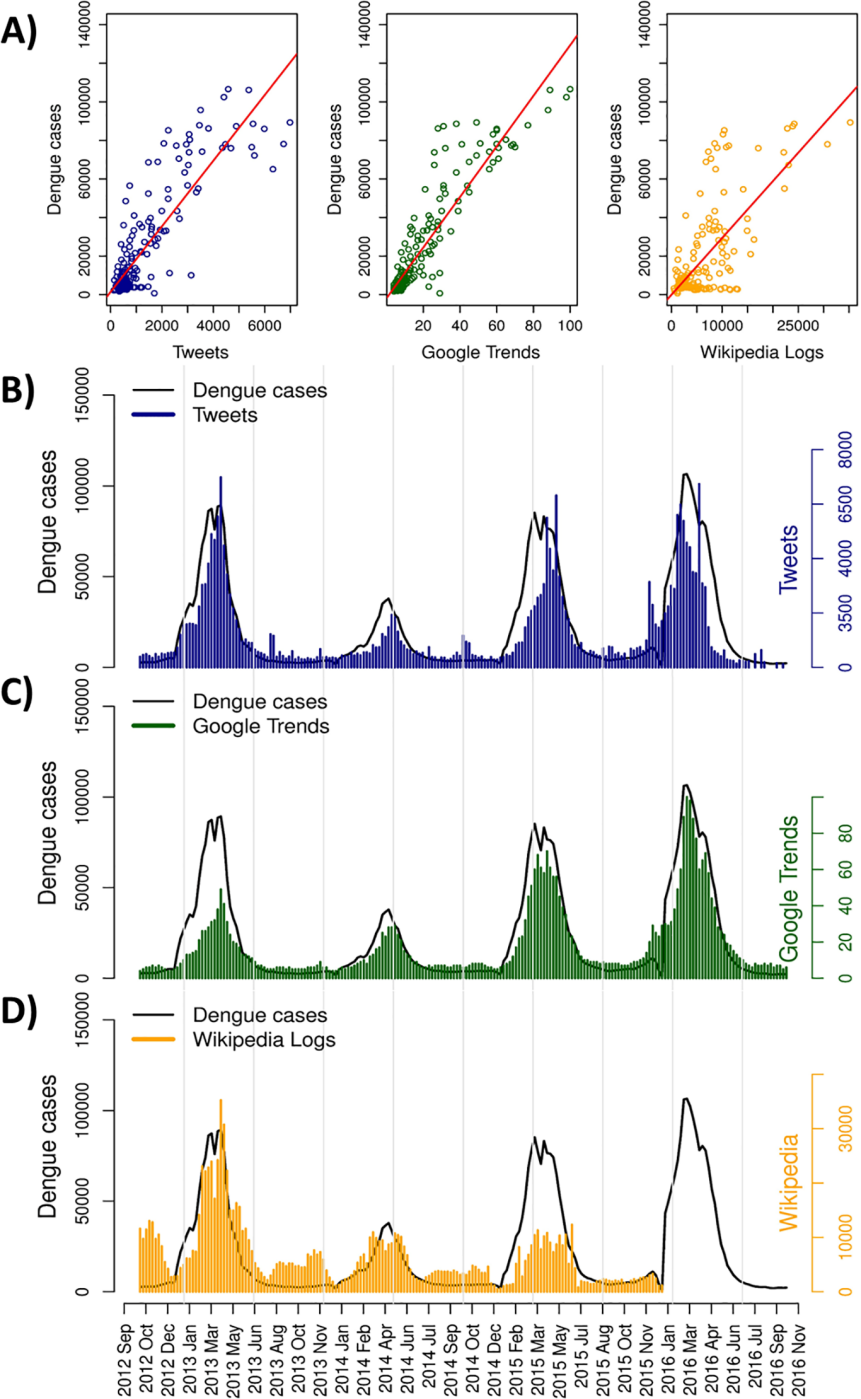
Country wide time series for web-based data and Dengue cases. Scatterplots and linear regression lines for all web-data analyzed: Tweets (r = 0.87, p<0.001), Google Trends (r = 0.92, p<0.001), and Wikipedia (r = 0.71, p<0.01) (A). Dengue Cases times series and association with Dengue web-data: Twitter data (B), Google Trends interest (C), and Wikipedia access logs (D). Each point on graph A represent data aggregate per week from September, 2012 through October, 2016.

We compared the time series of web-based data indicating Dengue activity with real observed Dengue cases in Brazil country level (Fig 1), between September, 2012 and October, 2016. Dengue cases occurred continuously with a high weekly variation, with a minimum of 694 cases per week. The highest incidence of Dengue was observed in the months of March and April of each year, reaching 106,558 cases per week (Fig 1). Tweets, Google Trends (GT) and Wikipedia access logs showed strong and positive association with the observed Dengue cases (Fig 1A). Tweets showed high variation, with an average of 1,213 tweets per week, ranging from 125 to 6,984 (Fig 1B). Tweets presented a high positive association with Dengue cases (r = 0.87, p<0.001), especially in 2013 and 2014 (Fig 1A and 1B). In the last trimester of 2015 (October to December), there was increased tweet activity not associated with Dengue.

The relative GT index was 17.51 on average, varying from 4 to 100 (Fig 1C). GT showed the stronger linear association with Dengue cases (r = 0.92, p<0.001), compared to tweets (Fig 1A). Wikipedia logs presented the smallest linear association (r = 0.71, p<0,01) with Dengue cases, but could also be considered high (Fig 1A). The values started at 517, and achieved 35,250 logs per week, with mean value of 6,481 (Fig 1D). Unfortunately, Wikipedia data was only available until December, 2015. There is important Wikipedia activity during non-epidemic periods not associated with real Dengue cases.

### Tweets are a useful tool for estimating Dengue occurrence at country level

**Fig 2:**
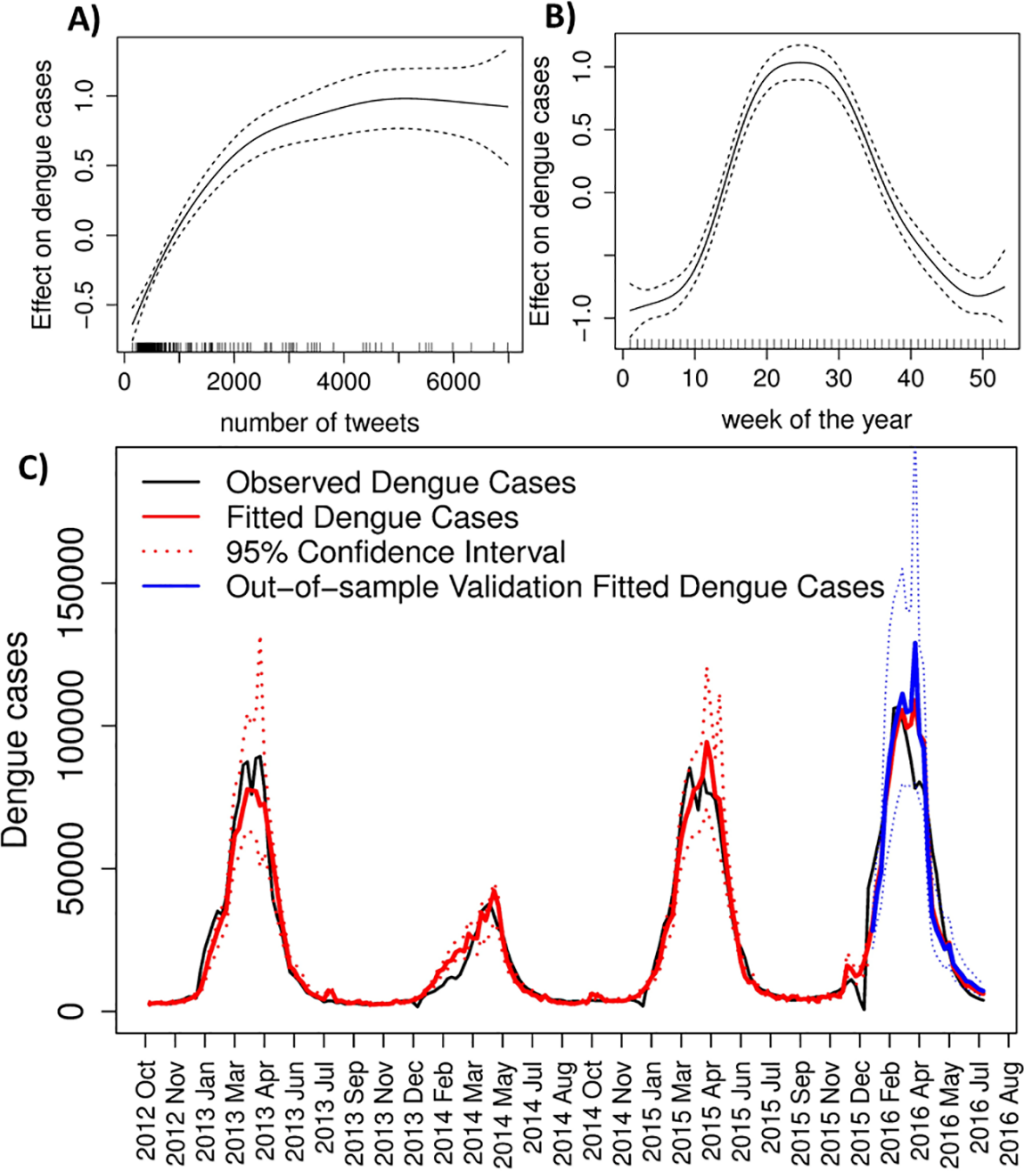
Tweets are a useful tool for estimating Dengue activity at country level. (A) The non-linear effect of Tweets on Dengue. (B) The non-linear effect of weeks on Dengue. (C) Time series of observed Dengue cases (black line); model in-sample estimated Dengue cases (red line), and out-of-sample estimated Dengue cases (blue line); and its 95% confidence interval (dashed red and blue lines) during 209 weeks.

Tweets with Dengue content were used to estimate weekly Dengue cases occurrence at country level, in Brazil (Fig 2, Table 2). Our selected model (Table 2) has tweets as covariate, as well as a temporal structure to account for the seasonality and annual cyclic characteristics of this disease. We can observe that the model with tweets plus a temporal structure presented a better Dengue estimation capacity than a model with either variables alone (Table 2, S2 Fig). We compared the selected tweets model with models including also “Dengue cases” as covariate. Three weeks is the usual time period for data from Dengue cases to become available [19], therefore we decided to include Dengue with three weeks lag (t-3) as explanatory variable for Dengue from week t. The latter model presented the best fit to observed data, with high explained deviance, low AIC, and reduced mean relative error (Table 2, S2 Fig). Otherwise, here the model with tweets and temporal structure was selected for further analyses, because it has the estimate capacity very similar to the model with Dengue as covariate, but is easy to apply and is also useful at city level (Table 2).

**Table 2:** Tweets are a useful tool for estimating Dengue activity at country level. Comparison between the selected model and other models with combinations of the variables: tweets, Dengue cases and temporal structures; using AIC, explained deviance and mean relative error as estimation capacity indicators.

Explanatory Variables	Model (Dengue ~ Negative Binomial (μ_t_, k))	AIC	Deviance Explained	Mean relative error *
Tweets + temporal structure + Dengue cases (three weeks lag)	log(μ_t_) = ƒ_**1**_(Tweets_t_) + Den_t-3_ + ƒ_**2**_(week_t_) + eyear_t_ + β_0_	3805.44	93.8	0.344
**Tweets + temporal structure** **(SELECTED MODEL)**	**log(μ**_**t**_**) = ƒ**_**1**_**(Tweets**_**t**_**) + ƒ**_**2**_**(week**_**t**_**) + eyear**_**t**_ **+ β**_**0**_	**3805.52**	**93.7**	**0.345**
Temporal structure + Dengue cases (three weeks lag)	log(μ_t_) = ƒ(week_t_) + eyear_t_ + Den_t-3_ + β_0_	3917.47	88.5	0.442
Temporal structure only	log(μ_t_) = ƒ(week_t_) + eyear_t_ + β_0_	3948.18	86.6	0.510
Tweets + Dengue cases (three weeks lag)	log(μ_t_) = ƒ(Tweets_t_) + Den_t-3_ + β_0_	4027.69	79.3	0.694
Tweets only	log(μ_t_) = ƒ(Tweets_t_) + β_0_	4103.40	69.7	0.954
Dengue cases (three weeks lag) only	log(μ_t_) = ƒ(Den _t-3_) + β_0_	4113.61	67.8	0.707

* mean absolute relative difference between predicted Dengue cases and observed cases

Our selected model indicates that tweets are a positive predictor for Dengue cases (Fig 2A), with an almost linear effect until 2,000 tweets, that stabilizes above this value. As expected, the relationship between Dengue and tweets is influenced by the week of the year (Fig 2B), since disease transmission is highly seasonal (Fig 1). Estimated Dengue cases showed a good fit to the observed data (Fig 2C), presenting a mean relative error of 0.345, and 93.7% of deviance explained by the model (Table 2). In order to validate the estimation capacity of our model, we applied out-of-sample analysis with tweets data not previously used by our model for adjustment. Our model could successfully estimate Dengue cases in this scenario, with the capacity for explaining the deviance of Dengue of 93,2% (Fig 2C).

### Dengue forecasting

**Fig 3:**
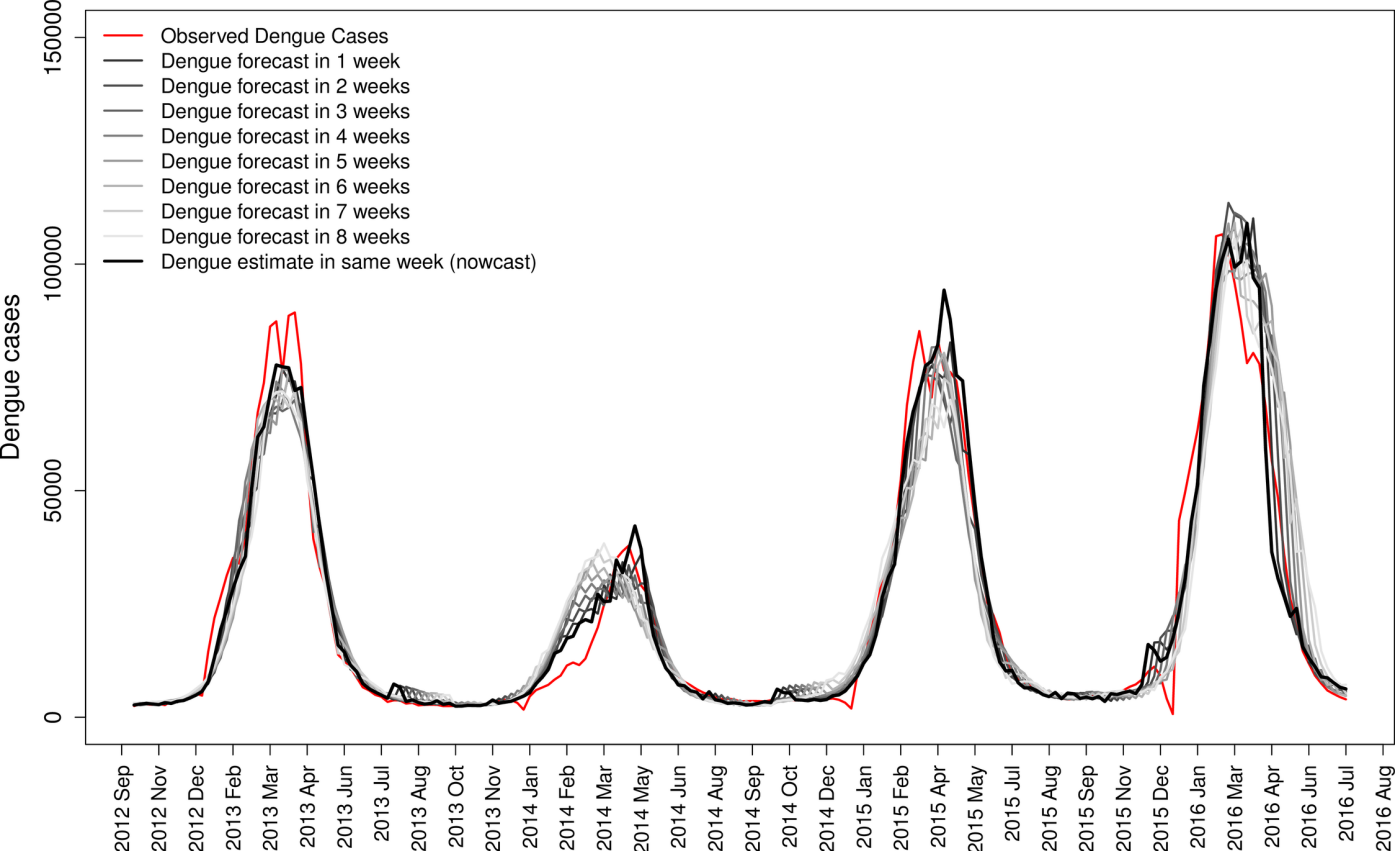
Forecast analysis. Capacity of the tweets to predict Dengue up to 8 weeks in advance. The model selected (Table 2) was adjusted to different time lags between tweets and Dengue cases. The lines indicate the model result of Dengue estimated in 1 to 8 weeks in advance of tweets.

Dengue forecasting, i.e. the prediction of the number of Dengue cases occurring in future weeks (up to 8 weeks), was also investigated (Fig 3, Table 3). The quality of the forecast varies with the week of prediction, as we can observe by the deviance explained index and the mean relative error of the prediction in relation to observed cases (Table 3). We also showed that tweets are performing better in estimating Dengue cases in the present week, “nowcast”, since people may tweet about the disease during its occurrence. Forecasting was possible with an increasing error with the increase in forecast weeks, but good approximation to real disease occurrence, as indicated by fitted and observed lines in the time series for four different epidemic years (Fig 3, Table 3).

**Table 3:** Forecast analysis. Dengue estimation capacity of tweets up to 8 weeks in advance. The model selected (Table 2) was adjusted to different time lags between tweets and Dengue cases, the numbers (Tw_-1_, Tw_-2_,…Tw_-n_) indicates the number of weeks that tweets are considered before the week of Dengue prediction.

MODEL	Model (Dengue ~ Negative Binomial (μ_t_, k))	FORECAST (week in advance)	AIC	Deviance Explained	R-sq. (adj)	Mean relative error *
Tw_t_	log(μ_t_) = ƒ_1_(Tweets_t_) + ƒ_2_(week_t_) + eyear_t_ + β_0_	0	3805.52	93.7	0.94	0.34
Tw_t-1_	log(μ_t_) = ƒ_1_(Tweets_t-1_) + ƒ_2_(week_t_) + eyear_t_ + β_0_	1	3825.92	93.0	0.94	0.36
Tw_t-2_	log(μ_t_) = ƒ_1_(Tweets_t-2_) + ƒ_2_(week_t_) + eyear_t_ + β_0_	2	3849.33	92.2	0.92	0.40
Tw_t-3_	log(μ_t_) = ƒ_1_(Tweets_t-3_) + ƒ_2_(week_t_) + eyear_t_ + β_0_	3	3868.49	91.4	0.90	0.44
Tw_t-4_	log(μ_t_) = ƒ_1_(Tweets_t-4_) + ƒ_2_(week_t_) + eyear_t_ + β_0_	4	3877.07	91.0	0.89	0.45
Tw_t-5_	log(μ_t_) = ƒ_1_(Tweets_t-5_) + ƒ_2_(week_t_) + eyear_t_ + β_0_	5	3881.43	90.8	0.88	0.40
Tw_t-6_	log(μ_t_) = ƒ_1_(Tweets_t-6_) + ƒ_2_(week_t_) + eyear_t_ + β_0_	6	3896.20	90.1	0.89	0.40
Tw_t-7_	log(μ_t_) = ƒ_1_(Tweets_t-7_) + ƒ_2_(week_t_) + eyear_t_ + β_0_	7	3918.60	88.9	0.88	0.44
Tw_t-8_	log(μ_t_) = ƒ_1_(Tweets_t-8_) + ƒ_2_(week_t_) + eyear_t_ + β_0_	8	3931.54	88.2	0.87	0.46

* mean absolute relative difference between predicted Dengue cases and observed cases

### Tweets signal can be obtained at city level

**Fig 4:**
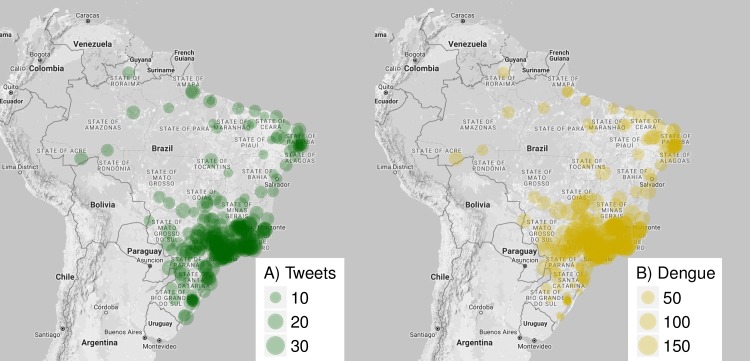
Tweets signal can be obtained at city level. Spatial distribution of evaluated cities in Brazil and their intensity of Dengue-related tweeting activity (A), and their incidence of Dengue cases (B). The data were aggregated from September, 2012 to October, 2016 and presented as cases or activity per 100,000 inhabitants.

Tweets were obtained from 283 different cities distributed all over Brazil, including all 5 regions and 26 states (Fig 4, S1 Table). We observe that cities with higher Twitter activity are mostly clustered at the southeastern region of the country (Fig 4A). These cities overlap with the region with the highest incidence of Dengue cases (Fig 4B).

### Tweets are a useful tool for estimating and forecasting Dengue activity at city level

**Fig 5:**
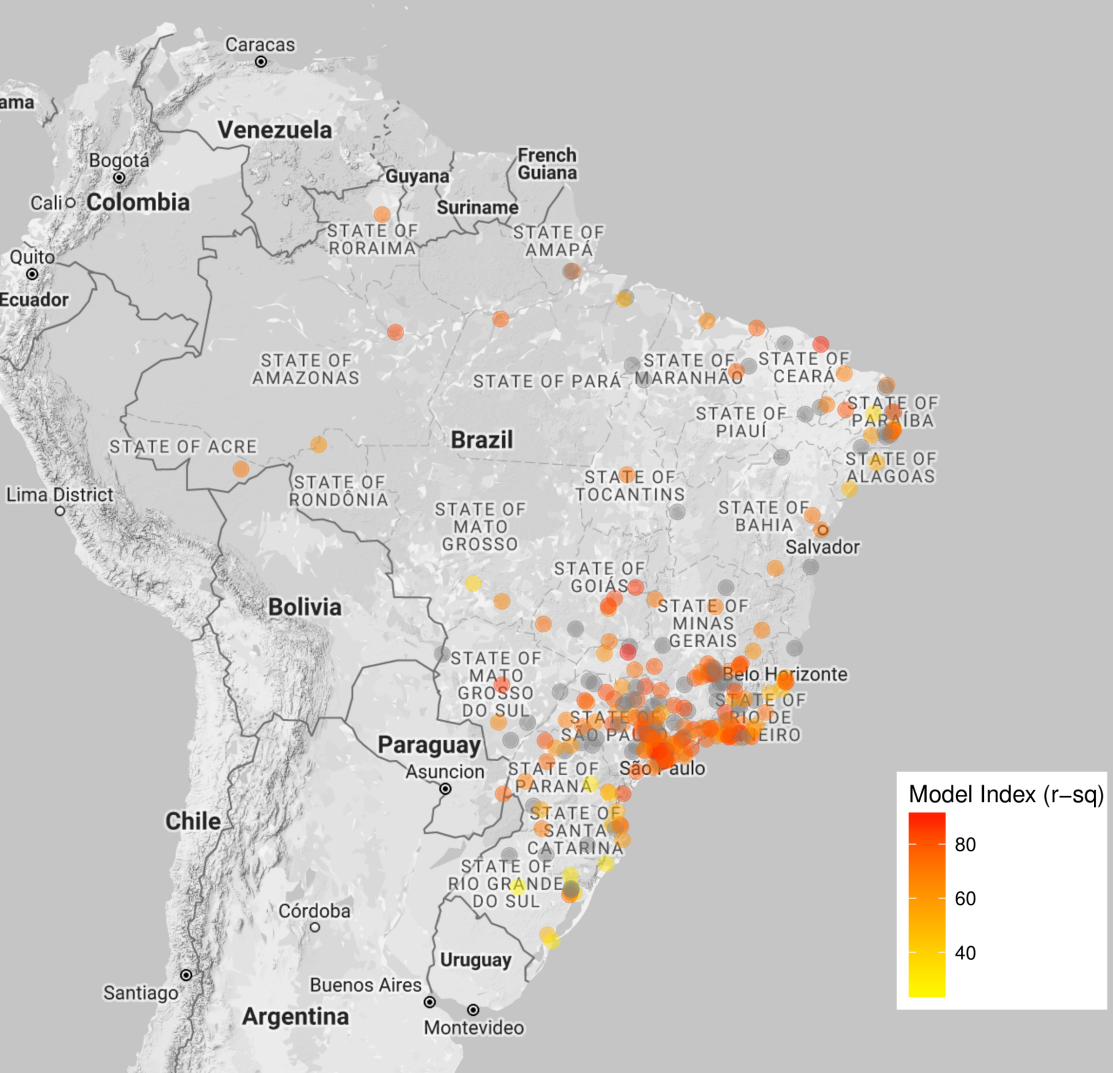
Heat map indicating the goodness-of-fit of the tweets model at city level. Deviance explained index resulting from the prediction model is shown. Cities that had too few data to be analyzed by the model are represented with grey circles. Cities with higher indices are mostly clustered at the southeastern region of the country.

We also analyzed the contribution of tweets to estimate Dengue at city level. For the majority of cities, we observed a high positive linear association between Dengue cases and tweets, with 67% of them with association above 50% (S1 Table). Our tweets model (Table 2) could successfully fit and estimate Dengue cases in 199 cities of a total of 283, since some cities had too few data for model estimate convergence. Model goodness-of-fit was high for most cities, with Dengue deviance explained above 60% in 88% of cities analyzed (Table 4, S1 Table). Cities with high Dengue estimation quality of our model are distributed around the country but mostly concentrated at the southeastern region (Fig 5).

**Table 4:** Dengue estimation capacity by tweets. Frequency distribution of cities considering the deviance explained result of the model applied to each city.

Goodness of fit of the Dengue estimation model of tweets at city level		
Deviance Explained	Frequency	
**>90%**	7	3.5%
**80–90%**	57	28.5%
**70–80%**	73	37%
**60–70%**	38	19%
**50–60%**	10	5%
**<50%**	14	7%
**Total**	199	100%

**Fig 6:**
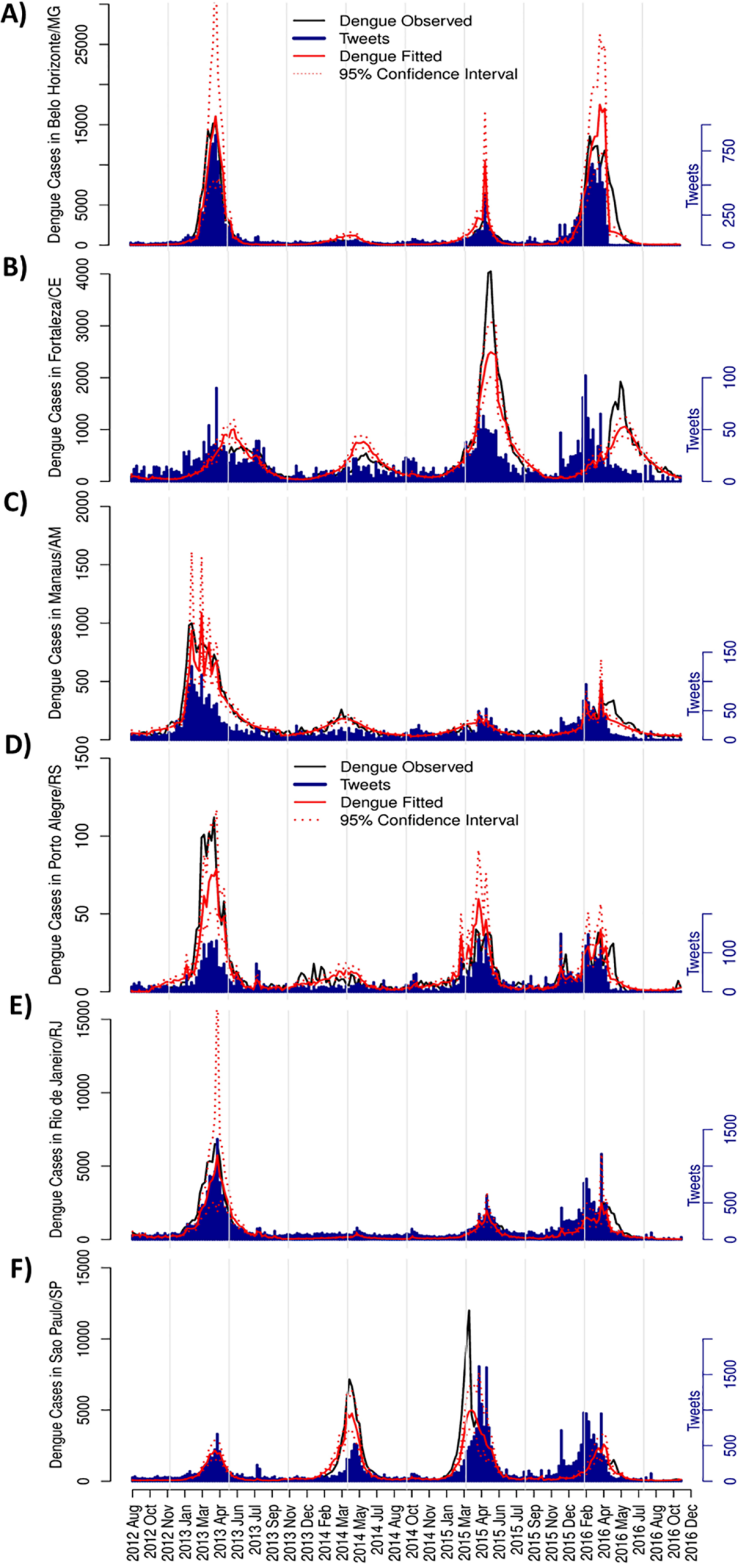
Dengue estimation by tweets model at city level. Time series with observed Dengue cases (black lines), tweets data (blue bars), model fitted Dengue cases (red lines) and 95% confidence interval (red dashed lines) at city level. (A) Belo Horizonte (r = 0.93, r^2^ = 90.3); (B) Fortaleza (r = 0.41, r^2^ = 90.0); (C) Manaus (r = 0.78, r^2^ = 83.5); (D) Porto Alegre (r = 0.71, r^2^ = 76.1); (E) Rio de Janeiro (r = 0.80, r^2^ = 82.6); and (F) São Paulo (r = 0.47, r^2^ = 89.0).

We selected cities in different regions of the country to further investigate and validate the model application for Dengue estimation at city level (Fig 6, S1 Table). We selected the following cities: Belo Horizonte (Fig 6A), Fortaleza (Fig 6B), Manaus (Fig 6C), Porto Alegre (Fig 6D), Rio de Janeiro (Fig 6E), and São Paulo (Fig 6F). In all cities, tweets successfully estimated Dengue cases, as shown by the approximation of observed Dengue cases and its predicted values by the model, and by the high values of deviance explained (ranging from 76.1% to 90.3%) (Fig 6, S1 Table). The model was also able to fit Dengue cases in cities with lower linear correlation indexes, as Fortaleza and Sao Paulo (Fig 6B and 6F, S1 Table). Important to notice that here we evaluated the Dengue estimation capacity of the same tweets model applied to country level, but each city would have improved results with models considering specific characteristics of each individual city.

### Dengue estimation capacity of tweets at city level is influenced by social factors

Tweets with Dengue content and their association with Dengue cases may be influenced by different factors. We divided the 283 cities into two groups, according to the quality of their Dengue estimation by the model: high quality group included cities (161) with model explained deviance equal or higher than 60%, and low quality group cities (122) with model explained deviance smaller than 60%, or zero (model did not converge). Cities with high quality of Dengue estimation based on the tweets model have a higher population, more Dengue cases and tweets activity (Table 5). They also had higher human development indices: mean (IDHM), education (IDHME) and income (IDHMI). Only longevity index (IDML) was not different between groups (Table 5). As expected, cities with good fit by the model were those with high coverage of houses with access to a personal computer and internet (Table 5).

**Table 5:** Goodness-of-fit of the tweets model is influenced by disease incidence, access to computers, internet and social factors. Cities were divided into two groups: high, for cities with model Dengue estimate explained deviance equal or higher than 60%, and, low, otherwise.

	Goodness-of-fit of the tweet model				
Variable Description	High		Low		p value
	Mean	Std Error	Mean	Std Error	
Population (n)	375,440.00	48,784.00	31,3882.00	10,1736.00	***
Gross Domestic Product per capita	34,599.00	1,793.00	28,611.00	1,844.00	**
IDHM Mean (2010)	0.77	0.003	0.74	0.004	***
IDHM Income (2010)	0.,75	0.004	0.73	0.005	*
IDHM Longevity (2010)	0.85	0.002	0.84	0.002	
IDHM Education (2010)	0.70	0.004	0.67	0.005	***
Houses with personal computer (%)	52.46	0.79	47.10	1.13	**
Houses with internet (%)	42.96	0.80	37.48	1.01	***
Dengue cases, 2012 (n)	333.90	54.31	125.20	28.71	**
Dengue cases, 2013 (n)	6,419.00	1,093.00	1,553.00	255.10	**
Dengue cases, 2014 (n)	2,465.00	391.50	1412.00	511.00	**
Dengue cases, 2015 (n)	6369.00	868.40	2667.00	730.10	**
Dengue cases, 2016 (n)	4,293.00	1,084.00	1,506.00	305.50	**
Total Dengue cases (n)	19,880.00	2,851.00	7,164.00	1,669.00	**
**Total Dengue Incidence (per 100,000 inhabitants)**	6,604.00	400.80	3,976.00	355.90	***
Tweets, 2012 (n)	38.65	9.22	23.08	8.84	***
Tweets, 2013 (n)	378.90	94.69	134.20	56.58	***
Tweets, 2014 (n)	136.30	27.17	94.70	48.06	***
Tweets, 2015 (n)	316.10	60.03	218.20	129.70	***
Tweets, 2016 (n)	288.50	72.19	147.10	74.02	***
Total Tweets (n)	1,159.00	256.30	586.80	301.00	***
**Twitter activity per 100,000 inhabitants**	270.00	13.14	137.00	7.01	***
Linear Correlation (Dengue~Tweets)	0.67	0.01	0.43	0.02	***
Weeks with tweets (%)	55.60	1.72	35.89	2.13	***
Weeks with Dengue (%)	85.82	1.11	67.23	2.57	***
**Mean relative error of model estimate**	1.43	0.06	0.98	0.11	***

* p value <0.01

** p value <0.001

***p value <0.0001

**Fig 7:**
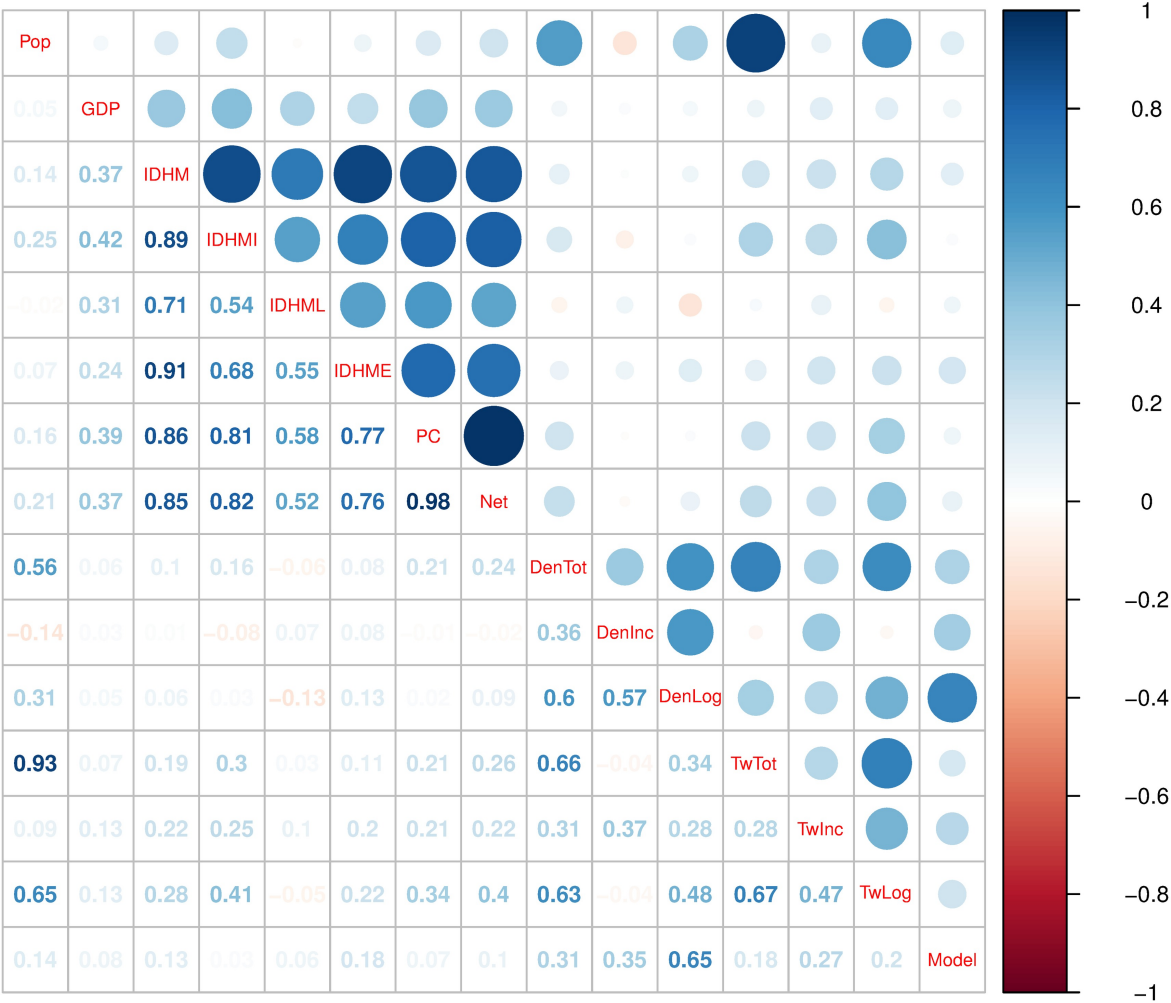
Correlation matrix. Correlation between different possible explanatory factors and the goodness-of-fit (explained deviance) of the final model. The variables are: population (Pop), gross internal product *per capita* (GDP), mean human development index (IDHM), human development index for income (IDHMI), human development index for longevity (IDHML), human development index for education (IDHME), coverage of houses with personal computer (PC), coverage of houses with internet access (Net), total Dengue cases (DenTot), total Dengue incidence in cases per 100,000 inhabitants (DenInc), total Dengue cases in logarithmic scale (DenLog), total tweets (TwTot), total tweets incidence in activity per 100,000 inhabitants (TwInc), total tweets in logarithmic scale (TwLog), and deviance explained by the model (Model). Total tweets and Dengue cases were calculated as the sum of occurrences from September, 2012 to October, 2016.

Otherwise, considering a linear regression association between the variables analyzed here (Table 5) and the Dengue estimate explained deviance by the model, we can observe that Dengue estimation capacity of tweets is strongly associated with Dengue incidence, but are not or weakly associated with population and development indexes (Fig 7).

## Discussion

In this study, we analyzed the potential of Twitter data for estimating and forecasting Dengue cases. Here we show that tweets are strongly associated with Dengue cases, and contribute not only for estimating, but also for forecasting Dengue activity up to 8 weeks in the future.

Tweets, Google Trends and Wikipedia access logs with Dengue content show a strong and positive association with officially registered Dengue cases in Brazil. However, during the last trimester of 2015, there was an important increase of tweets activity that was not associated with Dengue cases. This increase may be associated with an increase in Dengue tweeting activity that may have been caused by media news and the onset of the Zika epidemic in the country. The Zika virus is transmitted by the same vector as Dengue, the mosquito *Aedes aegypti*, and the disease's first symptoms are very similar, but serious complications include Guillain-Barré syndrome, and congenital infections can occur which may lead to microcephaly and maculopathy [33]. Zika, which was widely spread in the Pacific islands, was introduced in Brazil in 2014–2015 and caused a widespread epidemic in Latin America [33,34]. Google Trends, similar to Twitter, increased at the last trimester of 2015, indicating probably higher public concern with both diseases.

Twitter is a real-time source of information on Dengue symptoms activity in a population, and was shown to have strong correlation with the number of notified cases [21], however, that association may be stronger during the increasing and decreasing phases, than during the disease peaks. Twitter, as a social network, may indicate the need for the Dengue patient to notify the disease to colleagues, therefore being a good estimator of disease occurrence. Otherwise, the other web-based data available for Dengue and evaluated here, GT and Wikipedia, are based on search queries, which would indicate a potential interest or curiosity over the disease, being more subjected to marketing campaigns and confusion with other diseases.

Models built on the fraction of Google search volume for Dengue-related queries were previously shown to adequately estimate true Dengue activity in different seasons [8, 22]. Here we confirm the high association between Google Trends and Dengue disease, also useful for disease surveillance and prevention. Wikipedia data suffer from a variety of instabilities that need to be understood and compensated for [2]. Language as a location proxy can only be used in some cases, since it is impossible to be used at finer scale, or even to indicate exactly the country, an important limitation. Overall, our feeling is that all three sources of data are probably useful to estimate Dengue at country-wide level. However, amongst these three web-based data, tweets with personal experience provide a strong association with real disease with potential to be an important explanatory variable for Dengue estimation models both in country and city level.

The epidemiology of Dengue fever is highly seasonal, with multi-annual fluctuations, caused by the irregular circulation of its four serotypes, and the interplay between environmental drivers [35,36]. We built a simple model based on tweets together with a temporal structure that could successfully be used to estimate Dengue activity at country level, with 93.7% of explained deviance. The capacity of tweets to *nowcast*, i.e. predict the present events as they occur, may be already enough to provide a time advantage to understanding Dengue situation moment. Twitter was also useful in similar way for tracking and forecasting behavior in the influenza-like illness, as a measure of public interest or concern [11]. Dengue forecast was also possible using the model with tweets as covariate, with up to 8 weeks or 2 months of forecasting window. This result suggests that Twitter data can be used in the development of a proactive surveillance program and help health managers to better directed their resources for disease prevention.

One advantage of Twitter is that it can be geolocated at city level, which is a useful spatial resolution for surveillance. This feature strongly differentiates it from other available web-based data, such as Google Trends and Wikipedia [3,9]. While GT are available per state [22], the Wikipedia logs can only be aggregated per language [2]. Cities with higher tweets activity are those with higher Dengue incidence. Both Dengue occurrence and Twitter use are usually associated with cities with higher concentration of population or urbanization [3,14]. Similarly, GT and Dengue cases correlate better in states with higher Dengue incidence [22].

The Twitter data has also some limitations to be considered. Not everyone who submits a tweet with Dengue content is actually ill, but just interested or curious about it. Good surveillance will depend on a sufficient volume of interest to generate signals and compensate noise [8]. Therefore, a main challenge remains at areas with smaller population of Twitter users [4]. The tweets model performed better in areas with high Dengue incidence, but its performance was only weakly associated with population size and development index. This may suggest a robust model that can successfully be applied to smaller and less developed cities, which would improve the application effectiveness of the model as a surveillance tool. One advantage of including tweets into forecast models is to improve real-time estimations of Dengue incidence, overcoming difficulties of traditional Dengue surveillance systems that rely solely on case report data. Twitter captures information from individuals, especially at earlier stages of illness, who may search health information on the internet before or even instead of making medical visits, and publish this knowledge to seek help and comfort from friends. Tweets-based models may actually be even more useful in endemic regions of the world where the traditional surveillance system is too weak and slow to react to disease notification.

Here we show that the high Dengue estimation capacity of tweets model is influenced by human development indices and internet access. Important to observe that mean, education and income development indices which is associated with more houses with access to a personal computer and internet are also associated with tweets incidence. Otherwise, longevity development index is less associated with tweets incidence and activity, suggesting that young and adults may be the majority of users of this data. The accuracy of Google Trends was not found to be strongly influenced by socio-economic factors, particularly because it relies on internet searches, which may be robust enough to capture population-level disease dynamics [22]. Despite these social limitations, it is clear that tweets-based surveillance provides adequate citywide and countrywide Dengue estimates. Social factors, however, may limit the value of using tweets to examine epidemics within a city. At this stage, freely available tweet data are not sufficient to provide accurate determination of space within a specific city.

The capacity of tweets to estimate Dengue cases represents a valuable complement to assist traditional Dengue surveillance. A novel data source, like Twitter, could complement traditional surveillance at low-cost, since it is passive, free, and requires minimal resources to run [3,11]. These data can help reduce some of the many gaps that exist in Dengue surveillance methods, such as low sensitivity and accuracy, and timeliness [13,14,19]. Improving Dengue surveillance in a cost-effective way remains a major obstacle. In Brazil, the underreporting is about 50%, but can reach values as high as 90%, and the reporting delay is estimated to be approximately 3 to 4 weeks [19]. The main added benefit in monitoring social media behavior through tweets is the potential for early warning. Detecting and confirming results of prevention and control measures is possible at the interface between computer science, epidemiology, and medicine [4]. Our study therefore demonstrates that tweets are a web-based data that strongly associate with Dengue cases and have the potential to successfully estimate Dengue cases. Tweets are an easy to use, cost-effectiveness, useful and robust tool for estimating Dengue cases, both at country and city level, and for Dengue forecasting until 8 weeks in the future.

## Supporting information

S1 Fig Comparison between selected and discarded models to explain the relation between tweets and Dengue. Selected generalized addictive model (gam) residual and distribution analyses (A), and other discarded models with fitted Dengue estimation capacity demonstrated in (B): linear regression model, generalized additive mixed model with and without autoregressive components, and generalized linear models with and without autoregressive components.
(TIF)

S2 Fig Comparison of the selected model with other model candidates. Different combination of the variables tweets, Dengue and temporal structures were evaluated. Graphic demonstration of estimated and observed Dengue cases in 209 weeks period. A) Model with only Tweets. B) Model with only temporal structure. C) Model with only Dengue cases with 3 week of lag or delay. D) Model with temporal structure and Dengue with 3 weeks of lag. E) Model with tweets and Dengue with 3 weeks of lag. F) Model with the three variables: tweets, temporal structure and Dengue with 3 weeks of lag.
(TIF)

S1 Table Detailed list of cities evaluated in the study (283) and its respective indexes. (XLSX)
