# Supplementary figures and images for: Dengue prediction by the web: Tweets are a useful tool for estimating and forecasting Dengue at country and city level
Source: PLoS Negl Trop Dis. 2017 Jul 18;11(7):e0005729. doi: 10.1371/journal.pntd.0005729 (PMC5533462; doi:10.1371/journal.pntd.0005729)

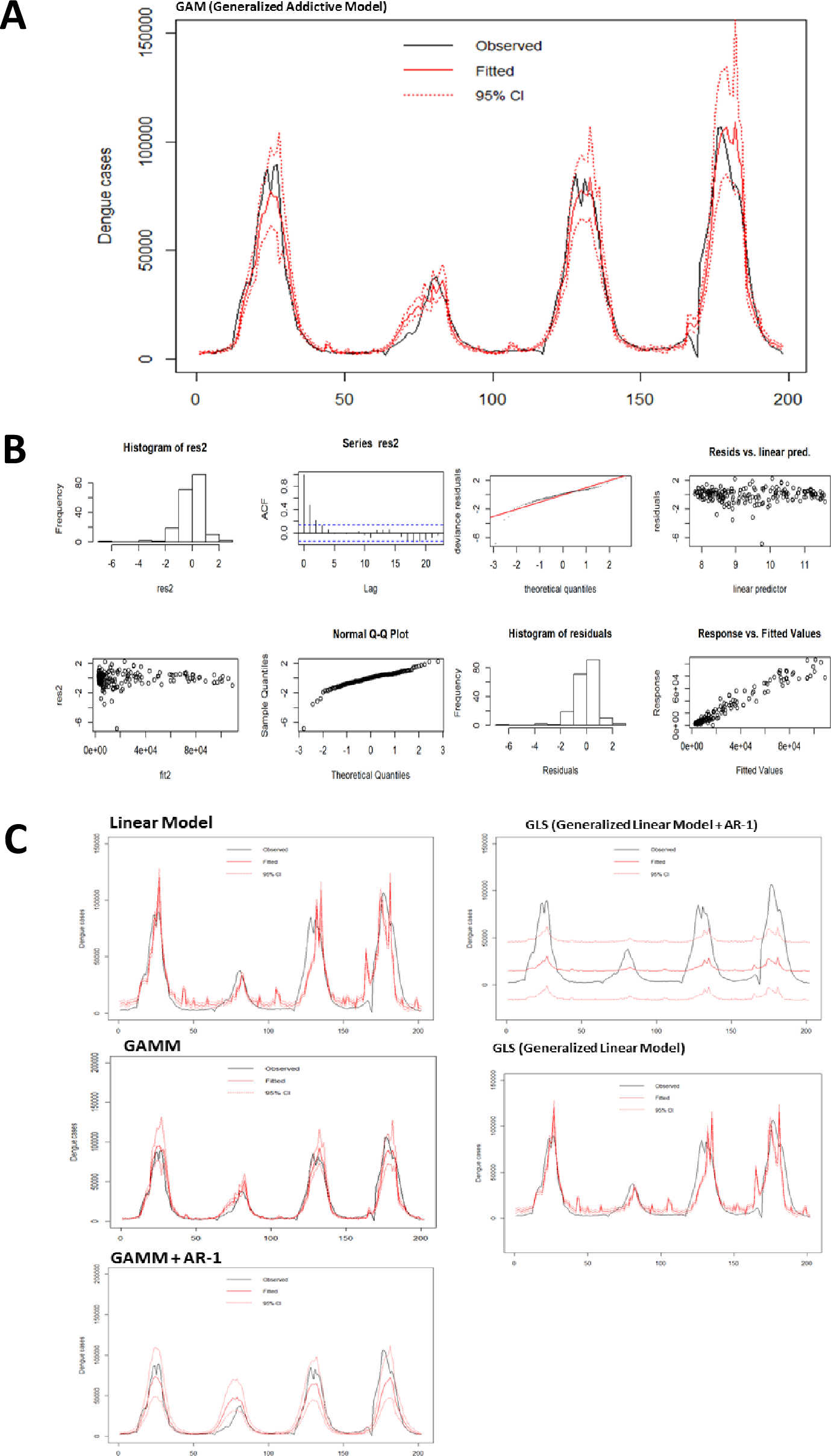

Supplement: S1 Fig — Selected generalized addictive model (gam) residual and distribution analyses (A), and other discarded models with fitted Dengue estimation capacity demonstrated in (B): linear regression model, generalized additive mixed model with and without autoregressive components, and generalized linear models with and without autoregressive components. (TIF) [file pntd.0005729.s001.tif]

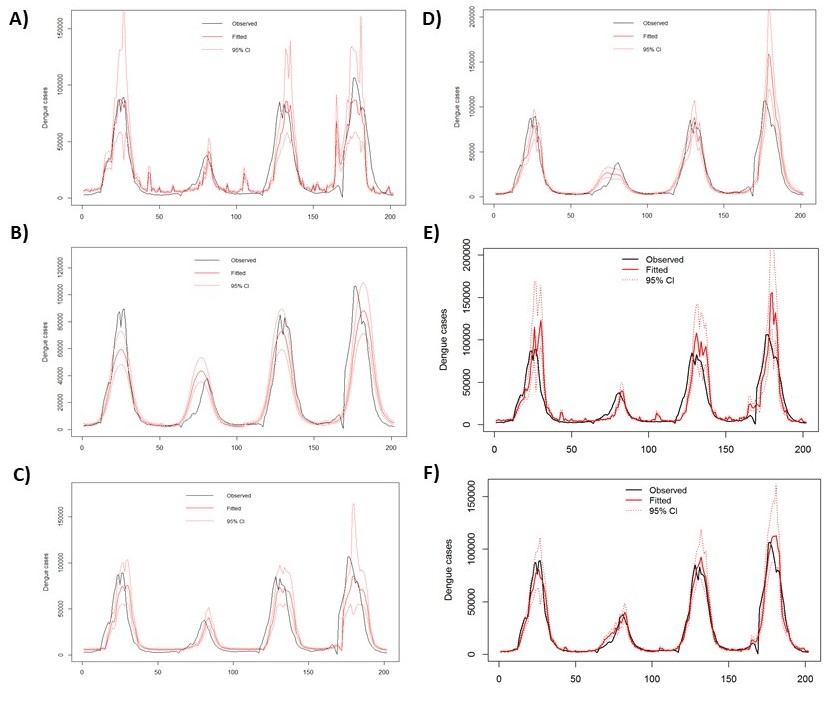

Supplement: S2 Fig — Different combination of the variables tweets, Dengue and temporal structures were evaluated. Graphic demonstration of estimated and observed Dengue cases in 209 weeks period. A) Model with only Tweets. B) Model with only temporal structure. C) Model with only Dengue cases with 3 week of lag or delay. D) Model with temporal structure and Dengue with 3 weeks of lag. E) Model with tweets and Dengue with 3 weeks of lag. F) Model with the three variables: tweets, temporal structure and Dengue with 3 weeks of lag. (TIF) [file pntd.0005729.s002.tif]
